# Supplementary material for: Development of a computer-aided design software for the quantitative evaluation of aesthetic damage
Source: PLoS One. 2019 Dec 18;14(12):e0226322. doi: 10.1371/journal.pone.0226322 (PMC6919621; doi:10.1371/journal.pone.0226322)
Supplement: S1 Code site — 1. (ZIP) [file pone.0226322.s001.zip › [EESC_jr][Projeto]Relatorio_07_21/reports/9/2017.02.04/15:26:48/report.pdf]

Preâmbulo

Na data de 10/01/1998 no horário 12:31, o Perito(a) Sr. Rafael Yonezawa de Mello, Estudante, designado(a)para proceder ao exame pericial solicitado em: João Gastão, do gênero , nascido(a) em 31/12/1969, ocupação , portador(a) do RG , cujo endereço , passa a expor a avaliação de dano estético corporal.

Exame objetivo da lesão

A lesão ora objeto de análise teve como natureza ocorrido em 31/12/1969. O prejuízo estético consiste em lesão cicatricial consolidada na região: no lado .

Trata-se de cicatriz Hipertrófica de tamanho/frequência Única: Retilínea de orientação Horizontal de pigmentação Normocrômica medindo centímetro(s) na horizontal, centímetros na vertical e milímetros de Profundidade. De contornos Regulares. Perceptivel em centímetros de distância nas relações íntimas. Perceptivel em metros nas relações sociais. A lesão tem repercução na vida diária conforme fotos anteriores e atuais..

Conclui-se que classificada segundo os parâmetros de dano estético AIPE - Análise do Impacto do Prejuízo Estético (escala de seis níveis e pontuação entre 0 e 50 pontos), como sendo dano estético relativo à área 1, a lesão é caracterizada por pontos e grau de prejuízo estético: Não relevante.

Escore final: ;
Grau de prejuízo: Não relevante.

, 10/01/1998

____

Sr. Rafael Yonezawa de Mello
